# Can Physical Activity While Sedentary Produce Health Benefits? A Single-Arm Randomized Trial

**DOI:** 10.1186/s40798-020-00278-3

**Published:** 2020-10-02

**Authors:** Marvin A. Sackner, Jose R. Lopez, Veronica Banderas, Jose A. Adams

**Affiliations:** 1 Mt. Sinai Medical Center of Greater Miami, Miami Beach, FL USA; 2 Sackner Wellness Products LLC, Miami, FL USA

**Keywords:** Physical activity, Physical inactivity, Sedentary, Oxygen consumption, METS, Gentle jogger

## Abstract

**Background:**

Sedentary time poses a risk to health. Substituting physical activity for inactivity is obvious but this requires a behavior change. Interventions advocated to decrease uninterrupted physical inactivity (defined as Metabolic Equivalent of Task (METS) less than 1.5) are important. One such intervention is accomplished with the Gentle Jogger (GJ), a low risk motorized wellness device which produces effortless, rapid motion of the lower extremities simulating locomotion or fidgeting. GJ produces health benefits in type 2 diabetes, heart disease, and high blood pressure. The purpose of this trial was to ascertain whether GJ increases METS above 1.5 to explain its effectiveness despite sedentary behavior or whether tapping is responsible.

**Methods:**

A randomized single-arm trial was conducted. Subjects were randomized to begin the study in either the supine or seated postures and on the same day crossed over with the starting posture reversed. Oxygen consumption was measured at rest and during GJ.

**Results:**

Twenty-six subjects were studied (15 women and 11 men) with a mean age of 44 ± 15 years and BMI 27.9 ± 5.0, 19 were overweight or obese, and 7 had normal BMI. GJ increased oxygen consumption and METS 15% in the seated posture and 13% in the supine posture. No individual receiving GJ achieved METS exceeding 1.5.

**Conclusions:**

In a moderately obese population, GJ in seated or supine posture did not exceed 1.5 METS. The values are comparable to those reported for sit-stand interventions and cannot explain the health benefits of GJ.

**Trial registration:**

ClinicalTrials.gov, NCT03602365. Registered on July 26, 2018

## Key Points

- Gentle jogger (GJ) provides health benefits without increasing oxygen consumption above 1.5 METS
- Avoidance of health risks of prolonged sitting can potentially be mitigated by passive simulation of locomotion.
- Harnessing health benefits with sustained endothelial shear stress using a non-invasive non-pharmacological intervention (GJ) may have major implications for public health.

## Introduction

Adults spend increasing amounts of waking time sedentary in seated, reclining, or lying postures expending low levels of energy amounting to less than 1.5 METS. For American adults, this constitutes an average of 55% of their waking time or 7.7 h/day, and for Europeans, an average of 40%. Sedentary time is an independent risk factor to health such that moderate to vigorous physical activity (MVPA) does not mitigate high levels of sedentary time, and adverse health effects tend to persist despite engaging in MVPA. These health risks include increased incidence of cardiovascular disease, stroke, hypertension, type 2 diabetes, chronic respiratory diseases, dementia, certain cancers, and premature mortality [1–4].

Viewing television programs while sitting is the most common daily activity apart from working and sleeping. In the USA, adults spend on average five hours daily viewing TV [5]. In a meta-analysis of over one million adults, high levels of sitting time increased the risk of premature mortality in all but the most physically active individuals who accumulated greater than 1 h/day of moderate-intensity physical activity [6]. To counteract the health risks of uninterrupted sitting in the workplace, the obvious solution is to substitute physical activity for inactivity which is easier said than done since less than 5 to 40% of American adults meet physical activity guidelines [7–9].

Uninterrupted sitting produces increases oxidative stress and endothelial dysfunction owing to reduced muscular activity of the lower extremities. Here, leg blood flow decreases, blood pools in the calves, arterial pressure increases, and shear stress (friction) to the vascular endothelium diminishes. The latter is particularly relevant because the reduced activity of endothelial nitric oxide synthase (eNOS), the gene stimulated by shear stress decreases the bioavailability of nitric oxide leading to a pro-oxidant and inflammatory milieu associated with the pathogenesis of type 2 diabetes, heart disease, hypertension, osteoporosis, sarcopenia, as well as endometrial, colon and lung cancer [10, 11].

Several interventions have been proposed to reduce prolonged, uninterrupted physical inactivity exemplified by excessive sitting in the home and workplace. These include breaking up sitting time with brief periods of standing or walking, desks that require positioning from seated to standing posture, self-monitoring, and counseling. None have shown consistent long-term adherence to postural changes or compliance with physical activity recommendations [12–18]. Because of low adherence to physical activity guidelines, an effective intervention must be effortless and independent of postural changes. The gentle jogger (GJ) is a low-risk wellness device that meets these requirements [General Wellness: Policy for Low Risk Devices Guidance for Industry and Food and Drug Administration Staff, 27 September 2019]. Here, FDA decided not to regulate low risk wellness devices and suggested the following claims: (1) as part of a healthy lifestyle, may help to reduce the risk of certain chronic diseases or conditions; and (2) as part of a healthy lifestyle, may help living well with certain chronic diseases or conditions. Chronic diseases and conditions for which a healthy lifestyle is associated with risk reduction or help in living well were identified as heart disease, high blood pressure, and type 2 diabetes which have been reported with health benefits attributed to the GJ [19–21]. Since GJ produces effortless movements of the lower extremities, we investigated the possibility that it increases physical activity, defined as any bodily movement that increases energy expenditure as estimated by oxygen consumption [22]. The latter can be converted to METs, signifying Metabolic Equivalent of Task at which the body expends energy during sitting at rest equivalent to 3.5 ml of oxygen consumed per kilogram of body weight per minute [23]. When this value is less than 1.5 while sitting or lying down, such behavior is considered sedentary. The current trial will determine as to whether GJ increases energy expenditure above 1.5 METS.

## Methods

### IRB Approval for Energy Expenditure Study

This investigation and its informed consent were approved by the Western Institutional Review Board (WIRB), Study Number: 11172318 and WIRB: 20170208374 (WIRB, Puyallup, WA 98374-2115). It was registered at ClinicalTrials.gov (NCT03602365) as a sub-unit of the larger protocol in which multiple postures were investigated. The current protocol was designed as a randomized single-arm trial in which subjects were randomized to begin the study in either the supine or seated postures and on the same day crossed over with the starting posture reversed. Data for this sub study was collected between September and November 2018. Randomization occurred using coin toss. (Supplemental File contains the CONSORT Study Flow Diagram)

### Participants

Twenty-six ambulatory individuals comprising 15 women and 11 men were recruited for this investigation by word of mouth and gave their informed consent to participate. Their mean age was 44 years SD 15 years and mean BMI of 27.9, SD 5.0. They were post-prandial for at least 3 h and asked not to drink coffee on the day prior to their participation. BMI was computed to characterize participants as follows: BMI normal weight 18.5–24.9, overweight 25–29.9, and obese 30 or more. For women, four had normal BMI, seven were overweight, and four were obese. For men, three had normal BMI, four were overweight, and four were obese. Studies were conducted in the mid-morning and early afternoon. Each subject was randomized to begin the study in either the seated or supine posture.

### Patient and Public Involvement

This study could not have been possible without the focus group participation of a group of more than 23 subjects of varying ages, physical and clinical characteristics (age, BMI, gender, healthy and diseased) who used the GJ and provided input and feedback to Sackner Wellness, as to their overall experience, ease of use and tolerability on the GJ. No participants were directly involved in the design or conduct of the study. Participants became involved in the research during the recruitment via word of mouth or seeing a volunteer recruitment poster. All subjects received financial remuneration for their participation. Participants were able to withdraw from the study at any time and without having to provide a reason. Since the study participants were recruited by word of mouth, most referred a friend or relative to join the study. Each study participant received an approved informed consent, and where given ample time to ask questions about the study. The study results were not compiled until completion of the entire cohort; therefore, subjects were not told about their individual results. The participants were made aware of their contribution of clinical data to research through their informed consents. After publication, dissemination of the results will be done via social media and scientific meetings. The cumulative information obtained in this study will be made available to all study participants and the general public.

### Gentle Jogger (GJ)

GJ has been described in previous publications [20, 21, 24, 25]. It incorporates microprocessor controlled, DC motorized movements of foot pedals placed within a chassis to repetitively tap against a semi-rigid surface for effortless simulation of locomotion while the subject is seated or lying in bed. Each time the moving foot pedals strike the bumper, a small pulse is added to the circulation as a function of pedal speed, which in the current study was set to ~ 190 steps in place [21]. This stepping rate is close to the intermittent taps 248 per minute of by voluntary fidgeting (1 min on and 4 min of rest) that improves endothelial dysfunction as reflected by a reduction of elevated blood pressure [26].

### Oxygen Consumption and Ventilation

Oxygen consumption was obtained over 10 min using the MetaCheck® system (KORR Medical Technologies, Salt Lake City, UT 84109). The MetaCheck is an indirect calorimeter which measures oxygen consumption (VO_2_) and estimates resting metabolic rate (RMR) based on the measured VO_2_ using an assumed respiratory quotient (R.Q.) of 0.83 [27, 28] (KORR, Medical Technologies, Salt Lake City UT 84109).

### Procedure

After instruction on breathing through a mouthpiece and application of a nose clip, subjects were instructed to rest without movement or talking in the posture selected, either seated on a padded chair or lying on a hospital bed in a quiet, light-dimmed room for 10 min. A mouthpiece and nose clip were applied, and they breathed to a hose connected to the MediCheck apparatus for 10 min. VO_2_ and ventilatory values were displayed by the MediCheck and transferred to an Excel spreadsheet. Another 10-min recording was carried out, and the mean of the two recording periods was taken as the value for the rest period. Two 10-min recordings were repeated during operation of GJ. The subjects were permitted to walk to a nearby rest room to “stretch their legs” and urinated if necessary. They then rested for 10 min in the opposite posture and the same procedure as the initial trial was repeated. The study could not be blinded, since the use of the GJ device is obvious. Additionally, since the study compared a baseline non-intervention posture for each subject to GJ use, data analysis could not be blinded. Statistical analysis was performed using Statistica (Statsoft, Tulsa, OK). Comparison between primary outcome oxygen consumption in each posture was performed using non-parametric Kruskal-Wallis. Data are expressed as mean and standard deviation.

## Results

Table 1 depicts the mean energy expenditure for the 15 women and 11 men in this study as reflected by oxygen consumption and METs. GJ increased these parameters by about 15% in the seated posture and about 13% in the supine posture in our moderately obese tested population. No individual receiving GJ reached METs that exceeded 1.5. Women and men showed similar energy expenditure values for GJ (Tables 2). GJ increased minute ventilation mostly as the result of increased tidal volume rather than the respiratory rate (Table 3). None of the subjects reported any discomfort or inability to remain seated or supine while using the GJ

**Table 1:** Energy expenditure for all 26 subjects

	Seated resting	Seated GJ	%Increase	Supine resting	Supine GJ	%Increase
**Oxygen consumption** (ml/min)	190 ± 31	215 ± 35*	14	188 ± 33	213 ± 34*	13
**Oxygen consumption** (ml/min/kg)	2.5 ± 0.4	2.9 ± 0.6*	16	2.5 ± 0.5	2.8 ± 0.5*	12
**MET**	0.93 ± 0.13	1.07 ± 0.14*	15	0.92 ± 0.11	1.04 ± 0.12*	13
**Energy expenditur**e (Kcal/min)	0.92 ± 0.14	1.03 ± 0.17*	12	0.91 ± 0.17	1.02 ± 0.16*	12

*Legend*: Energy expenditure for all subjects expressed as mean and standard deviation(±SD) in seated and supine posture. The resting posture was compared to gentle jogger (GJ) in the same posture. There was a statistically significant increase from resting in oxygen consumption, MET, and energy expenditure (*p ˂ 0.001 GJ vs resting)

**Table 2:** Energy expenditure for females and males

	Seated resting	Seated GJ	%Increase	Supine resting	Supine GJ	%Increase
**Females**						
**Oxygen consumption** (ml/min)	175 ± 26	197 ± 25*	14	172 ± 28	194 ± 26*	13
**Oxygen consumption** (ml/min/kg)	2.5 ± .4	2.9 ± .6*	17	2.4 ± .4	2.8 ± .6*	16
**MET**	.95 ± .13	1.09 ± .16*	15	.92 ± .11	1.04 ± .14*	12
**Energy expenditure** (Kcal/min)	.85 ± .12	.94 ± .12**	11	.85 ± .18	.92 ± .42†	8
**MALES**						
**Oxygen consumption** (ml/min)	212 ± 23	243 ± 29*	15	214 ± 22	240 ± 22*	12
**Oxygen consumption** (ml/min/kg)	2.5 ± .4	2.8 ± .4*	15	2.5 ± .4	2.8 ± .5*	11
**MET**	.92 ± .11	1.02 ± .11**	12	.91 ± .12	1.02 ± .11*	13
**Energy expenditure** (Kcal/min)	1.02 ± .10	1.17 ± .14*	15	1.03 ± .11	1.15 ± .10*	12

*Legend:* Energy expenditure for female and male subjects expressed as mean and standard deviation(±SD) in seated and supine posture. The resting posture was compared to gentle jogger (GJ) in the same posture. There was a statistically significant increase from resting oxygen consumption, MET and energy expenditure in both females and male subjects (*p ˂ 0.001 GJ vs resting; **p ˂ 0.01; †*p* ˃ 0.05)

**Table 3:** Ventilation for all 26 subjects

	Seated resting	Seated GJ	%Increase	Supine resting	Supine GJ	%Increase
**Tidal volume** (ml)	510 ± 120	660 ± 250*	29	470 ± 100	590 ± 170**	26
**Minute ventilation** (L)	5.50 ± 1.05	7.30 ± 2.39*	33	5.32 ± 1.10	6.82 ± 1.33*	28
**Respiratory rate per minute**	11.7 ± 2.9	12.4 ± 1.6	6	11.8 ± 3.0	12.9 ± 2.8†	10

*Legend:* Ventilation parameters for all subjects expressed as mean and standard deviation(±SD) in seated and supine posture. The resting posture was compared to gentle jogger (GJ) in the same posture. There was a statistically significant increase from resting tidal volume and minute ventilation in both posture during use of GJ (*p ˂ 0.001 GJ vs resting; **p ˂ 0.01, †*p* ˂ 0.05)

## Discussion

### Energy Expenditure

In recent years, there have been several publications addressing the health hazards of sedentary behavior as manifested by uninterrupted, prolonged sitting in the context of the growing prevalence of obesity and health risks. Interventions to reduce workplace sitting time have included utilization of treadmill desks, stepping devices, pedaling workstations, counseling, mindfulness training, prompts for periodic breaks to standing and walking. For the long term, adherence has been disappointing. Active physical pedaling workstations do not reduce sitting time at work compared to counseling alone [17, 29–31]. Sit-stand desks have been a commercial success owing to the expectation that standing significantly increases energy expenditure reflected by changes in oxygen consumption due to contraction of leg muscles to maintain such posture. In our review of 15 studies of sit-stand desks comprising almost 500 subjects, approximately equally divided among men and women, mean age 30 years, range 22 to 48 years, mean standing oxygen consumption was only 9% greater than sitting with a range from 4 to 12% [30, 32–47]. GJ increased seated oxygen consumption by about 15% with no significant difference between men and women. Therefore, the modest extra energy expenditure of standing or during seated GJ operation cannot be expected to produce significant weight loss even as a long-term outcome. The small increase of ventilation with standing over sitting of 12% [48] is less than the 33% increase for seated GJ but cannot produce significant health benefits.

### Gentle Jogger and Health Benefits

The beneficial health effects of GJ relate to its increase of pulsatile endothelial shear stress (friction) as found for its predicate device, the motion platform [49]. The fabrication of the latter was based upon the pioneering experiments in a perfused, isolated blood vessel by Hutcheson and Griffith [50]. With a pump that delivered pulses of 2 mmHg constant amplitude with a constant flow of a physiological solution, they found that nitric oxide was released from the endothelium of a perfused blood vessel with the peak response between 250 and 360 pulses per minute. We confirmed their observations utilizing an isolated porcine aorta perfused with a physiological solution of (1) non-pulsatile flow, (2) pulsatile flow at 60 pulses per minute, and (3) pulsatile flow superimposed with added pulses from a motion platform moving at 180 times per minute. The latter moved the perfused aorta back and forth repetitively in the ‘z plane’ to add pulses to the perfusate as a result of fluid inertia. With pulsatile flow, nitric oxide as measured by an ISO-NO electrode increased about 300% above steady flow and showed a further increase with additional pulses generated by the activity of the motion platform about 1000% over non-pulsatile flow). In anesthetized pigs, placed on the motion platform and moved at 180 times per minute a significant decreases of mean femoral blood pressure from 94 mmHg at baseline to 80 mmHg after 30 min of treatment with the motion platform occurred with corresponding mean pulmonary artery pressure decreases from 12 mmHg to 10 mmHg. This effect was attenuated by L-NAME, a nitric oxide inhibitor, demonstrating that the vasodilator properties of pulsatile shear stress to the endothelium are consistent with increased nitric oxide bioavailability [51]. Operation of the motion platform is called whole body periodic acceleration (WBPA or pGz).

### Vascular Effects of WBPA

WBPA acutely increased microvascular blood flows as assessed by injection of colored microspheres (median diameter 15 microns) into the circulation of swine, viz., epicardium (71%), endocardium (93%), cerebrum (183%), brain stem (177%), renal cortex (53%), ileal mucosa (69%), gastric antral mucosa (72%), and liver (86%). These blood flows returned to baseline 10 min after discontinuation of WBPA except in myocardium where blood flow remained significantly elevated [52]. In normal subjects and patients with chronic diseases, the motion platform increased nitric oxide bioavailability at rest and exercise [53, 54]. WBPA increased brachial flow-mediated dilatation, a measure of endothelial function related to nitric oxide bioavailability in healthy and sedentary adults [55–57]. It acutely increased coronary flow reserve in healthy subjects and patients with coronary artery disease [58]. WBPA increased walking distance and quality of life in patients with stable ischemic heart disease and peripheral vascular disease [59]. In patients with angina, 20 sessions of WBPA decreased left ventricular end-diastolic volume index by 18% and increased ejection fraction from 50 to 55% [60]. Despite positive vascular effects, the motion platform that was not widely adopted for human applications because it was too expensive, limited to application solely to the supine posture, and non-portable owing to its large footprint and weight (211 kg).

### Gentle Jogger

Owing to structural limitations of the motion platform, a new device called the gentle jogger (GJ) was fabricated that produced sustained pulsatile shear stress to the endothelium as a low-cost, portable improvement over its predicate motion platform. The GJ produces the same effects as WBPA while transmitting pulses throughout the vascular system remote from foot tapping. The suggested usage duration of GJ is 30 min, one to three times daily, but longer durations such as 1 h or greater are safe, beneficial, and without deleterious effects. GJ can be self-administered in home and workplace, and, provide effortless health benefits without resort to multitasking. Thus far, clinical trials of its operation have been completed for high blood pressure, heart disease, and type 2 diabetes, all of which have various degrees of endothelial dysfunction.

### High Blood Pressure

Physical inactivity during uninterrupted sitting or lying in bed is rapidly associated with increases of systolic and diastolic arterial blood pressures. The rise of blood pressure begins 5 min after baseline while seated, and 10 min after baseline while supine and slowly continues over a 40-min observation period. In the seated posture, for control, peak change of systolic pressure was 7.5 mmHg *above* the baseline, and for GJ, peak change of systolic pressure was 8.4 mmHg *below* the baseline. This finding demonstrates that GJ is an effective, technology to acutely reduce elevated blood pressure associated with prolonged sitting presumably related to its capability of releasing the vasodilator mediator, endothelial nitric oxide. This study dealt with its acute effects of GJ, but long-term studies are needed to establish chronic effectiveness in the workplace and home where uninterrupted sitting watching television is often the norm.

### Heart Disease

Heart rate variability (HRV) reflects the neural balance between sympathetic and parasympathetic autonomic nervous systems (ANS). Reduced HRV is a marker of ANS dysfunction that occurs in diabetes, coronary artery disease, heart failure, hypertension, aging, and frailty. Thirty minutes of GJ significantly increased time-domain measures and Poincare parameters of HRV in both seated and supine postures as measured from electrocardiographic analysis. Since low HRV indicates compromised health [61], GJ usage is promoted improved cardiac fitness.

### Type 2 Diabetes

Physical inactivity is a high-risk factor for type 2 diabetes and increased physical activity improves indices of glycemic control. Continuous glucose monitoring (CGM) provides investigation of glycemic control during activities of daily living. A pilot study was undertaken to determine the effects of the portable GJ on glycemic indices of healthy and type 2 diabetes subjects using CGM 22 volunteers (11 type 2 diabetics and 11 healthy subjects), using continuous glucose monitoring (CGM) for 14 days. In healthy subjects, there were significantly lower values of mean glucose (mGlu) and SUM compared to BL for all days of GJ usage. In type 2 diabetics, mGlu, SUM, and area under the curve (AUC) were significantly lower compared to BL, for all days of GJ usage and post GJ. Time above range (TAR, glucose > 181 mg/dl) was significantly lower, and time in range (TIR, glucose 70–180 mg/dl) significantly improved during GJ in type 2 diabetics without change in the coefficient of variability (%CV). Therefore, GJ has a potential role in type 2 diabetes management [19].

## Study Limitation

The current study was not specifically designed to determine the mechanisms of action of GJ. Investigators have used passive leg movement as a means to increase vascular shear stress and have shown a threefold increase in blood flow which is nitric oxide dependent [62], enhanced interstitial vascular endothelial growth factor (VEGF), along with increased genomic expression of eNOS [63–65] and furthermore have advocated for the use of passive limb movement as an approach to asses nitric oxide mediated vascular function [66]. Based on our previous work with the predicate device WBPA, GJ also induces pulsations to the circulation thereby inducing the beneficial effects of sustained pulsatile shear stress to the vascular endothelium. A priori, we did not define a specific study population or composition since our goal was to test the effects of GJ on oxygen consumption in both genders. Coincidentally, the recruited study population was younger but skewed towards overweight to obese females and males. Although the effects of GJ on METS in older healthy subjects might be even less, the study composition in the present study is a particularly vulnerable population for sedentary behavior.

## Conclusion

Application of GJ does not alter sedentary inactivity as estimated by METS but its foot tapping properties as simulated locomotion induces health benefits.

## Additional File

**Additional file 1.** CONSORT Study Flow Diagram.

## Data Availability

The data used to support the findings of this study are available from the corresponding author upon request.

## Abbreviations

ANS: Autonomic nervous system
AUC: Area under the curve
BMI: Body mass index
CGM: Continuous glucose monitoring
eNOS: Endothelial nitric oxide synthase
FDA: Food and Drug Administration
GJ: Gentle jogger
HRV: Heart rate variability
mGlu: Mean glucose
METS: Metabolic equivalent of task
MVPA: Moderate to Vigorous Physical Activity
pGz: Periodic Acceleration in the z plane (a.k.a WBPA)
RMR: Resting metabolic rate
R.Q: Respiratory quotient
VO_2_: Oxygen consumption
WBPA: Whole body periodic acceleration

## References

[1] Agosti V, Graziano S, Artiaco L, Sorrentino G (2009). Biological mechanisms of stroke prevention by physical activity in type 2 diabetes. Acta Neurol Scand.

[2] Booth FW, Roberts CK, Thyfault JP, Ruegsegger GN, Toedebusch RG (2017). Role of Inactivity in Chronic Diseases: Evolutionary Insight and Pathophysiological Mechanisms. Physiol Rev.

[3] Patterson R, McNamara E, Tainio M, de Sa TH, Smith AD, Sharp SJ (2018). Sedentary behaviour and risk of all-cause, cardiovascular and cancer mortality, and incident type 2 diabetes: a systematic review and dose response meta-analysis. Eur J Epidemiol.

[4] Wajngarten M, Silva GS (2019). Hypertension and Stroke: Update on Treatment. Eur Cardiol.

[5] Grontved A, Hu FB (2011). Television viewing and risk of type 2 diabetes, cardiovascular disease, and all-cause mortality: a meta-analysis. JAMA..

[6] Ekelund U, Steene-Johannessen J, Brown WJ, Fagerland MW, Owen N, Powell KE (2016). Does physical activity attenuate, or even eliminate, the detrimental association of sitting time with mortality? A harmonised meta-analysis of data from more than 1 million men and women. Lancet..

[7] Craighead DH, Heinbockel TC, Hamilton MN, Bailey EF, MacDonald MJ, Gibala MJ (2019). Time-efficient physical training for enhancing cardiovascular function in midlife and older adults: promise and current research gaps. J Appl Physiol (1985).

[8] Keadle SK, McKinnon R, Graubard BI, Troiano RP (2016). Prevalence and trends in physical activity among older adults in the United States: A comparison across three national surveys. Prev Med.

[9] Troiano RP, Berrigan D, Dodd KW, Masse LC, Tilert T, McDowell M (2008). Physical activity in the United States measured by accelerometer. Med Sci Sports Exerc.

[10] Derbre F, Gratas-Delamarche A, Gomez-Cabrera MC, Vina J (2014). Inactivity-induced oxidative stress: a central role in age-related sarcopenia?. Eur J Sport Sci.

[11] Katzmarzyk PT, Powell KE, Jakicic JM, Troiano RP, Piercy K, Tennant B (2019). Sedentary Behavior and Health: Update from the 2018 Physical Activity Guidelines Advisory Committee. Med Sci Sports Exerc.

[12] Bailey DP, Locke CD (2015). Breaking up prolonged sitting with light-intensity walking improves postprandial glycemia, but breaking up sitting with standing does not. J Sci Med Sport.

[13] Compernolle S, DeSmet A, Poppe L, Crombez G, De Bourdeaudhuij I, Cardon G (2019). Effectiveness of interventions using self-monitoring to reduce sedentary behavior in adults: a systematic review and meta-analysis. Int J Behav Nutr Phys Act.

[14] Gao L, Nguyen P, Dunstan D, Moodie M. Are Office-Based Workplace Interventions Designed to Reduce Sitting Time Cost-Effective Primary Prevention Measures for Cardiovascular Disease? A Systematic Review and Modelled Economic Evaluation. Int J Environ Res Public Health. 2019;16(5). 10.3390/ijerph16050834 PMC6427179 30866495

[15] Kruse NT, Hughes WE, Benzo RM, Carr LJ, Casey DP (2018). Workplace Strategies to Prevent Sitting-induced Endothelial Dysfunction. Med Sci Sports Exerc.

[16] Shrestha N, Grgic J, Wiesner G, Parker A, Podnar H, Bennie JA (2019). Effectiveness of interventions for reducing non-occupational sedentary behaviour in adults and older adults: a systematic review and meta-analysis. Br J Sports Med.

[17] Shrestha N, Kukkonen-Harjula KT, Verbeek JH, Ijaz S, Hermans V, Pedisic Z (2018). Workplace interventions for reducing sitting at work. Cochrane Database Syst Rev.

[18] Thorsen IK, Johansen MY, Pilmark NS, Jespersen NZ, Brinklov CF, Benatti FB (2019). The effect of frequency of activity interruptions in prolonged sitting on postprandial glucose metabolism: A randomized crossover trial. Metabolism.

[19] Adams JA, Banderas V, J. LR, Sackner MA. Portable Gentle Jogger Improves Glycemic Indices in Type 2 Diabetic and Healthy Subjects Living at Home: A Pilot Study. J Diabetes Res. 2020. 10.1155/2020/8317973 PMC7081036 32215273

[20] Adams JA, Patel S, Lopez JR, Sackner MA (2018). The Effects of Passive Simulated Jogging on Short-Term Heart Rate Variability in a Heterogeneous Group of Human Subjects. J Sports Med (Hindawi Publ Corp).

[21] Sackner MA, Patel S, Adams JA (2019). Changes of blood pressure following initiation of physical inactivity and after external addition of pulses to circulation. Eur J Appl Physiol.

[22] Caspersen CJ, Powell KE, Christenson GM (1985). Physical activity, exercise, and physical fitness: definitions and distinctions for health-related research. Public Health Rep.

[23] Jette M, Sidney K, Blumchen G (1990). Metabolic equivalents (METS) in exercise testing, exercise prescription, and evaluation of functional capacity. Clin Cardiol.

[24] Adams JA, Banderas V, Lopez JR, Sackner MA (2020). Portable Gentle Jogger Improves Glycemic Indices in Type 2 Diabetic and Healthy Subjects Living at Home: A Pilot Study. J Diabetes Res.

[25] Sackner M, Adams JA. Does Fibromyalgia Sit in a Chair? Symptomatic Relief with a Simulated Jogging. Fibromyalgia, Open Access. 2017.

[26] Morishima T, Restaino RM, Walsh LK, Kanaley JA, Fadel PJ, Padilla J (2016). Prolonged sitting-induced leg endothelial dysfunction is prevented by fidgeting. Am J Physiol.

[27] Miles-Chan JL, Sarafian D, Montani JP, Schutz Y, Dulloo AG (2014). Sitting comfortably versus lying down: is there really a difference in energy expenditure?. Clin Nutr.

[28] Richardson HB. The respiratory quotient. Physiol Rev. 1929;9.

[29] Hamilton MT, Hamilton DG, Zderic TW (2007). Role of low energy expenditure and sitting in obesity, metabolic syndrome, type 2 diabetes, and cardiovascular disease. Diabetes..

[30] Levine JA, Schleusner SJ, Jensen MD (2000). Energy expenditure of nonexercise activity. Am J Clin Nutr.

[31] Shrestha N, Bhaumik S (2015). Are interventions to reduce sitting at workplace effective?. J Family Med Prim Care.

[32] Amaro-Gahete FJ, Sanchez-Delgado G, Alcantara JMA, Martinez-Tellez B, Acosta FM, Merchan-Ramirez E (2019). Energy expenditure differences across lying, sitting, and standing positions in young healthy adults. PLoS One.

[33] Judice PB, Hamilton MT, Sardinha LB, Zderic TW, Silva AM (2016). What is the metabolic and energy cost of sitting, standing and sit/stand transitions?. Eur J Appl Physiol.

[34] McAlpine DA, Manohar CU, McCrady SK, Hensrud D, Levine JA (2007). An office-place stepping device to promote workplace physical activity. Br J Sports Med.

[35] Monnard CR, Miles-Chan JL (2017). Energy Cost of Standing in a Multi-Ethnic Cohort: Are Energy-Savers a Minority or the Majority?. PLoS One.

[36] Popp CJ, Tisch JJ, Sakarcan KE, Bridges WC, Jesch ED (2016). Approximate Time to Steady-state Resting Energy Expenditure Using Indirect Calorimetry in Young, Healthy Adults. Front Nutr.

[37] Roemmich JN (2016). Height-Adjustable Desks: Energy Expenditure, Liking, and Preference of Sitting and Standing. J Phys Act Health.

[38] Beers EA, Roemmich JN, Epstein LH, Horvath PJ (2008). Increasing passive energy expenditure during clerical work. Eur J Appl Physiol.

[39] Burns J, Forde C, Dockrell S (2017). Energy Expenditure of Standing Compared to Sitting While Conducting Office Tasks. Hum Factors.

[40] Caljouw SR, de Vries R, Withagen R (2017). RAAAF's office landscape The End of Sitting: Energy expenditure and temporary comfort when working in non-sitting postures. PLoS One.

[41] Carter SE, Jones M, Gladwell VF (2015). Energy expenditure and heart rate response to breaking up sedentary time with three different physical activity interventions. Nutr Metab Cardiovasc Dis.

[42] Cox RH, Guth J, Siekemeyer L, Kellems B, Brehm SB, Ohlinger CM (2011). Metabolic cost and speech quality while using an active workstation. J Phys Act Health.

[43] Creasy SA, Rogers RJ, Byard TD, Kowalsky RJ, Jakicic JM (2016). Energy Expenditure During Acute Periods of Sitting, Standing, and Walking. J Phys Act Health.

[44] Fountaine CJ, Johann J, Skalko C, Liguori GA (2016). Metabolic and Energy Cost of Sitting, Standing, and a Novel Sitting/Stepping Protocol in Recreationally Active College Students. Int J Exerc Sci.

[45] Gibbs BB, Kowalsky RJ, Perdomo SJ, Grier M, Jakicic JM (2017). Energy expenditure of deskwork when sitting, standing or alternating positions. Occup Med (Lond).

[46] Kanade AN, Gokhale MK, Rao S (2001). Energy costs of standard activities among Indian adults. Eur J Clin Nutr.

[47] Levine JA, Miller JM (2007). The energy expenditure of using a “walk-and-work” desk for office workers with obesity. Br J Sports Med.

[48] Gao Y, Silvennoinen M, Pesola AJ, Kainulainen H, Cronin NJ, Finni T (2017). Acute Metabolic Response, Energy Expenditure, and EMG Activity in Sitting and Standing. Med Sci Sports Exerc.

[49] Uryash A, Wu H, Bassuk J, Kurlansky P, Sackner MA, Adams JA (2009). Low-amplitude pulses to the circulation through periodic acceleration induces endothelial-dependent vasodilatation. J Appl Physiol (1985).

[50] Hutcheson IR, Griffith TM (1991). Release of endothelium-derived relaxing factor is modulated both by frequency and amplitude of pulsatile flow. Am J Phys.

[51] Adams JA, Moore JE, Moreno MR, Coelho J, Bassuk J, Wu D (2003). Effects of periodic body acceleration on the in vivo vasoactive response to N-omega-nitro-L-arginine and the in vitro nitric oxide production. Ann Biomed Eng.

[52] Adams JA, Mangino MJ, Bassuk J, Kurlansky P, Sackner MA (2001). Regional blood flow during periodic acceleration. Crit Care Med.

[53] Sackner MA, Gummels E, Adams JA (2005). Nitric oxide is released into circulation with whole-body, periodic acceleration. Chest..

[54] Sackner MA, Gummels E, Adams JA (2005). Effect of moderate-intensity exercise, whole-body periodic acceleration, and passive cycling on nitric oxide release into circulation. Chest..

[55] Matsumoto T, Fujita M, Tarutani Y, Yamane T, Takashima H, Nakae I (2008). Whole-body periodic acceleration enhances brachial endothelial function. Circ J.

[56] Takase B, Hattori H, Tanaka Y, Uehata A, Nagata M, Ishihara M (2013). Acute Effect of Whole-Body Periodic Acceleration on Brachial Flow-Mediated Vasodilatation Assessed by a Novel Semi-Automatic Vessel Chasing UNEXEF18G System. J Cardiovasc Ultrasound.

[57] Thijssen DH, Black MA, Pyke KE, Padilla J, Atkinson G, Harris RA (2011). Assessment of flow-mediated dilation in humans: a methodological and physiological guideline. Am J Physiol Heart Circ Physiol.

[58] Fukuda S, Shimada K, Kawasaki T, Kono Y, Jissho S, Taguchi H (2010). "Passive exercise" using whole body periodic acceleration: effects on coronary microcirculation. Am Heart J.

[59] Kohler M, Amann-Vesti BR, Clarenbach CF, Brack T, Noll G, Russi EW (2007). Periodic whole body acceleration: a novel therapy for cardiovascular disease. VASA Zeitschrift fur Gefasskrankheiten.

[60] Miyamoto S, Fujita M, Inoko M, Oba M, Hosokawa R, Haruna T (2011). Effect on treadmill exercise capacity, myocardial ischemia, and left ventricular function as a result of repeated whole-body periodic acceleration with heparin pretreatment in patients with angina pectoris and mild left ventricular dysfunction. Am J Cardiol.

[61] Dekker JM, Schouten EG, Klootwijk P, Pool J, Swenne CA, Kromhout D (1997). Heart rate variability from short electrocardiographic recordings predicts mortality from all causes in middle-aged and elderly men. The Zutphen Study. Am J Epidemiol.

[62] Mortensen SP, Askew CD, Walker M, Nyberg M, Hellsten Y (2012). The hyperaemic response to passive leg movement is dependent on nitric oxide: a new tool to evaluate endothelial nitric oxide function. J Physiol.

[63] Hellsten Y, Rufener N, Nielsen JJ, Hoier B, Krustrup P, Bangsbo J (2008). Passive leg movement enhances interstitial VEGF protein, endothelial cell proliferation, and eNOS mRNA content in human skeletal muscle. Am J Phys Regul Integr Comp Phys.

[64] Hoier B, Rufener N, Bojsen-Moller J, Bangsbo J, Hellsten Y (2010). The effect of passive movement training on angiogenic factors and capillary growth in human skeletal muscle. J Physiol.

[65] Trinity JD, Groot HJ, Layec G, Rossman MJ, Ives SJ, Morgan DE (2015). Passive leg movement and nitric oxide-mediated vascular function: the impact of age. Am J Physiol.

[66] Trinity JD, Groot HJ, Layec G, Rossman MJ, Ives SJ, Runnels S (2012). Nitric oxide and passive limb movement: a new approach to assess vascular function. J Physiol.

