# Supplementary figures and images for: Can Physical Activity While Sedentary Produce Health Benefits? A Single-Arm Randomized Trial
Source: Sports Med Open. 2020 Oct 2;6:47. doi: 10.1186/s40798-020-00278-3 (PMC7532252; doi:10.1186/s40798-020-00278-3)

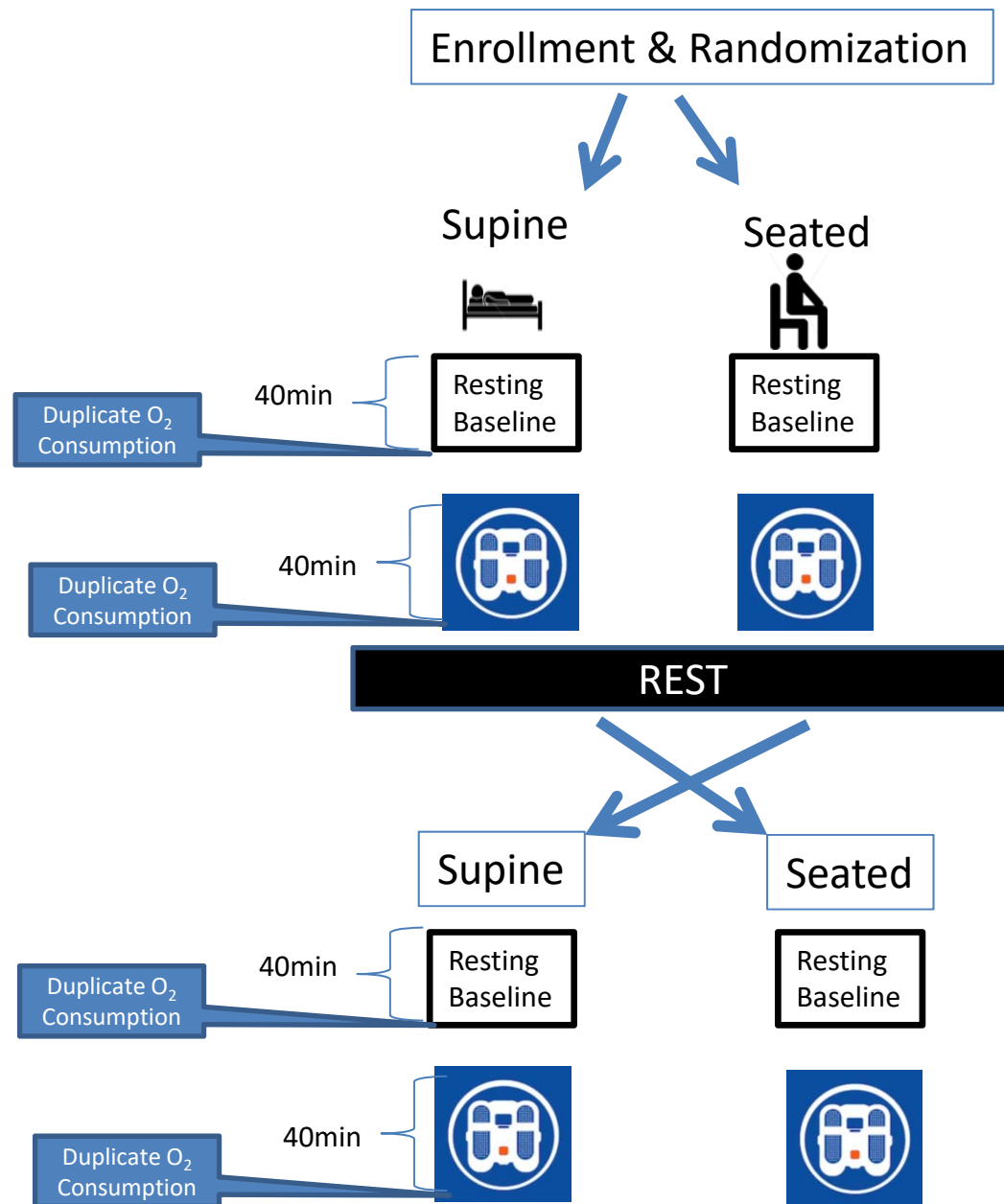

# CONSORT DIAGRAM

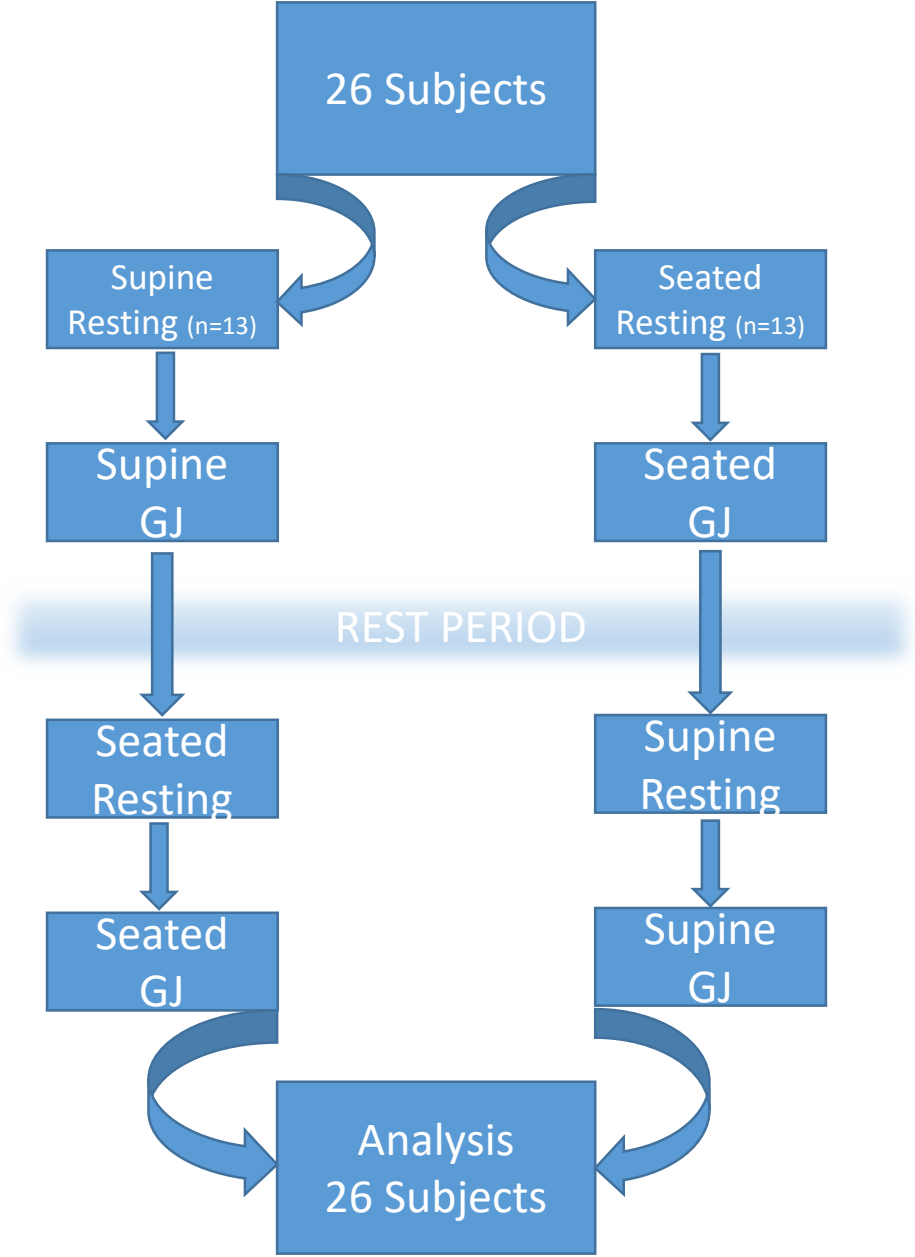

Supplement: Supplementary file 1 — Additional file 1. CONSORT Study Flow Diagram. [file 40798_2020_278_MOESM1_ESM.pdf]
